# A Pilot Study Assessing the Utility of Quantitative Myeloid-Derived Suppressor Cell Measurements in Detecting Posttraumatic Infection

**DOI:** 10.1097/CCE.0000000000001228

**Published:** 2025-03-17

**Authors:** Grant E. O’Keefe, Yiyang Wu, Nina Mirabadi, Minjun Apodaca, Qian Qui, Chihiro Morishima

**Affiliations:** 1 Department of Surgery, University of Washington, School of Medicine, Seattle, WA.; 2 Department of Laboratory Medicine and Pathology, University of Washington, School of Medicine, Seattle, WA.; 3 Department of Pediatrics and Harborview Injury Prevention Center, Seattle, WA.

**Keywords:** infection, myeloid-derived suppressor cells, sepsis, trauma

## Abstract

**OBJECTIVES:**

Biomarkers that facilitate earlier diagnosis of posttraumatic infection could improve outcomes by expediting treatment and mitigating complications, including sepsis. We hypothesized that circulating myeloid-derived suppressor cell (MDSC) counts could identify patients with posttraumatic infection.

**DESIGN, SETTING, AND PATIENTS:**

We conducted a single-center, prospective observational pilot study of trauma victims who required greater than or equal to 48 hours of mechanical ventilation. Whole blood was collected and tested by flow cytometry.

**INTERVENTIONS:**

None.

**MEASUREMENTS AND MAIN RESULTS:**

Samples were analyzed in real-time with an 11-parameter quantitative MDSC assay. Two physician adjudications of infection were performed through a blinded review of medical records. MDSC and other cell counts were compared between subjects with and without posttraumatic infection using non-parametric methods. Data are presented as medians (25th–75th percentile). The area under the receiver operating characteristic (ROC) curves were used to assess the accuracy of cell counts for diagnosing infection. Most subjects (*n* = 39) were male (79%) with a median age of 48 (interquartile range [IQR] 32–65), Injury Severity Score of 29 (IQR 21–41), and ICU length of stay of 13 days (IQR 8–19). Twenty-one (54%) developed an infection and 11 (28%) of the cohort died. We compared total MDSC (T-MDSC) counts closest to the day of infection diagnosis with the initial T-MDSC counts in subjects without infection. T-MDSC counts were higher in those with infection compared to those without infection (696 [368–974] and 304 [181–404] cells/μL, respectively; *p* < 0.001). Lymphocyte, neutrophil, and CD45+ leukocyte counts were not statistically different between the groups. The area under the ROC curve distinguishing those with infection from those without for T-MDSC was 0.83 (*p* < 0.001).

**CONCLUSIONS:**

MDSC counts determined by quantitative whole blood flow cytometrics can detect posttraumatic infection and may be useful to guide further diagnostic testing in critically ill trauma victims.

**KEY POINTS**

**Question:** Can circulating levels of myeloid-derived suppressor cells (MDSCs); measured by flow cytometry, detect infection in critically ill trauma victims?

**Findings:** In a cohort of 39 critically ill trauma patients, total-MDSC (T-MDSC) and polymorph nuclear-MDSC were higher in those who developed infection than those who did not. The area under the receiver operating characteristic curve distinguishing those with infection from those without for T-MDSC was 0.83. Elevated counts preceded the clinical diagnosis of infection in most cases.

**Meaning:** Measuring MDSCs by flow cytometry may be useful in the early detection of infection and in guiding further diagnostic evaluation.

Infection and sepsis contribute significantly to morbidity and mortality in hospitalized patients worldwide (1). Posttraumatic infection and sepsis develop in patients hospitalized for severe injuries and are typically identified toward the end of the first week of hospitalization when the patient is under close observation in an ICU (2, 3). Recent improvements in outcomes from sepsis have not occurred in trauma patients (4–7). Biomarkers that facilitate earlier detection of infection can potentially improve outcomes in these patients.

Myeloid-derived suppressor cells (MDSC) are a heterogeneous group of immature myeloid cells defined by their ability to potently suppress immune responses. Increased circulating MDSC levels are observed in several pathologic conditions including cancer, autoimmunity, and infection. MDSCs are thought to limit inflammation, help restore homeostasis, and increase during times of stress, such as with trauma (8). Polymorphonuclear-MDSCs, a subset of immature granulocytes are elevated in patients with infection (9, 10). In this pilot study, we evaluated whether a quantitative whole-blood MDSC assay could be used to help detect infection in patients admitted to a surgical ICU after traumatic injury (11). We hypothesized that total-MDSC counts (T-MDSCs) differ between subjects who develop infection and those who do not.

## MATERIALS AND METHODS

### Human Subjects and Study Enrollment

This single-center, prospective observational cohort study was approved by the University of Washington Institutional Review Board (STUDY00010131 [approval May 27, 2020] and STUDY00007918 [approval May 10, 2021]). All study procedures were conducted in accordance with the ethical standards of the University of Washington institutional review board and with the Helsinki Declaration of 1975. Strengthening the Reporting of Observational Studies in Epidemiology Guidelines for cohort studies were used and a checklist was included in the supplemental digital content (12). Subjects were identified according to previously described criteria for patients at risk for posttraumatic sepsis and prolonged ICU care (2, 13). We enrolled patients who were admitted to the surgical ICU at Harborview Medical Center (Seattle, WA), intubated and receiving mechanical ventilation for 48 hours, not expected to be extubated within the next 24 hours and when laboratory staff were available to perform flow cytometry (see additional details of enrollment and blood collection in **supplemental digital content,** http://links.lww.com/CCX/B474). Infection day assignments were based upon the review of clinical notes and laboratory results by two investigators (GO and NM) who were blinded to flow cytometry results and sepsis assignments followed the Sepsis-3 guidelines (1). Laboratory staff were blinded to clinical patient classification during testing. Six healthy subjects were enrolled as controls. The number of study subjects was not determined by power or sample size estimates. Did we end enrollment arbitrarily? To determine whether MDSC cell counts warranted further study.

### Flow Cytometry

Remnant blood samples drawn in K_2_EDTA vacutainer tubes (Becton Dickinson, Franklin Lakes, NJ) were analyzed within 4–12 hours of collection. We quantified circulating MDSC levels using a 9-color, 11-parameter flow cytometry assay that we developed (11). See **eMethods** and **eFigure 2** for additional details (http://links.lww.com/CCX/B474).

### Data Analyses

We present categorical data as counts and percentages and continuous data as medians with 25th–75th percentiles (i.e., interquartile range). Cell counts between subjects with and without infection were compared using the Mann-Whitney *U* test. Area under the receiver operating characteristic (ROC) curves are reported with 95% CIs. Data management and statistical analyses were performed using Stata, Version 15.1 (StataCorp, College Station, TX) and Prism Software, Version 10.1.1 (GraphPad, Boston, MA).

## RESULTS

### Study Subject Demographics, Injury Characteristics, and Outcomes

From June 12, 2021, to July 15, 2022, we enrolled 39 subjects who were predominantly male (*n* = 31; 79%) and sustained severe injuries to multiple body regions (Injury Severity Score 29 [(21–41)]). A total of 18 (46%) received a blood transfusion in the first 24 hours and 10 (26%) underwent an emergent laparotomy. **Table 1**, **eTable 1**, and **eFigure 1** (http://links.lww.com/CCX/B474) (enrollment diagram) contain additional details of the cohort.

**TABLE 1.** Study Demographics and Outcomes

Characteristics	*n* = 39
Age, median (IQR, yr)	48 (32, 65)
Sex, *n* (%)	
Female	8 (21%)
Male	31 (79%)
Race, *n* (%)	
American Indian or Alaska	3 (8%)
Asian	2 (5%)
Black	6 (15%)
White	25 (64%)
Not documented	3 (8%)
Mechanism of injury, *n* (%)	
Blunt	31 (79%)
Penetrating	8 (21%)
Injury Severity Score, median (IQR)	29 (21, 41)
Body regions abbreviated injury scale score ≥ 3, *n* (%)^a^	
Head	20 (51%)
Neck	7 (18%)
Chest	22 (56%)
Abdomen	11 (28%)
Spine	12 (31%)
Lower extremity	11 (28%)
Initial first systolic blood pressure measured in the emergency department < 90 mm Hg, *n* (%)	5 (13%)
RBC transfusion first 24 hr, *n* (%)	
None	21 (54%)
1–4 units	11 (28%)
5–9 units	2 (5%)
10+ units	5 (13%)
Emergency laparotomy, *n* (%)	10 (26%)
Outcomes	
Hospital length of stay, median (IQR, d)	22 (16, 46)
ICU length of stay, median (IQR, d)	13 (8, 19)
Duration of mechanical ventilation, median (IQR, d)	10 (6, 18)
Infection, n (%)	21 (54%)
Sepsis or septic shock	11 (28%)
Died, n (%)	11 (28%)

IQR = interquartile range.

a Body regions with frequency < 10% are not shown which include the face, upper extremity, and external injuries.

Continuous data are presented as median (IQR) and discrete data as *n* (%).

### Utility of MDSC Counts for the Detection of Infection

We examined flow cytometry data from timepoints closest to, and when possible, preceding infection diagnosis. Infection occurred at a median of 7 days after admission (range, 3–11 d), with MDSC counts analyzed from these patients obtained at a median of 6 days after ICU admission (range, 2–8 d). For 12 of 21 infected patients (57%), sample collection preceded the documented infection day by 1–4 days. Eight of the remaining nine subjects had blood obtained on the day of infection diagnosis, whereas one had their sample obtained 1 day after diagnosis. Ventilator-associated pneumonia (19/21) was the most common infection with the remainder being bacteremia and a surgical site infection. For the 18 subjects without infection, we used MDSC counts from the first blood sample collected after study enrollment (median 3.5 d after admission, range, 2–8 d). A total of 29 of the 39 subjects had 2 or more samples tested, and serial MDSC counts are shown in **eFigure 3** (http://links.lww.com/CCX/B474).

**Figure 1.**
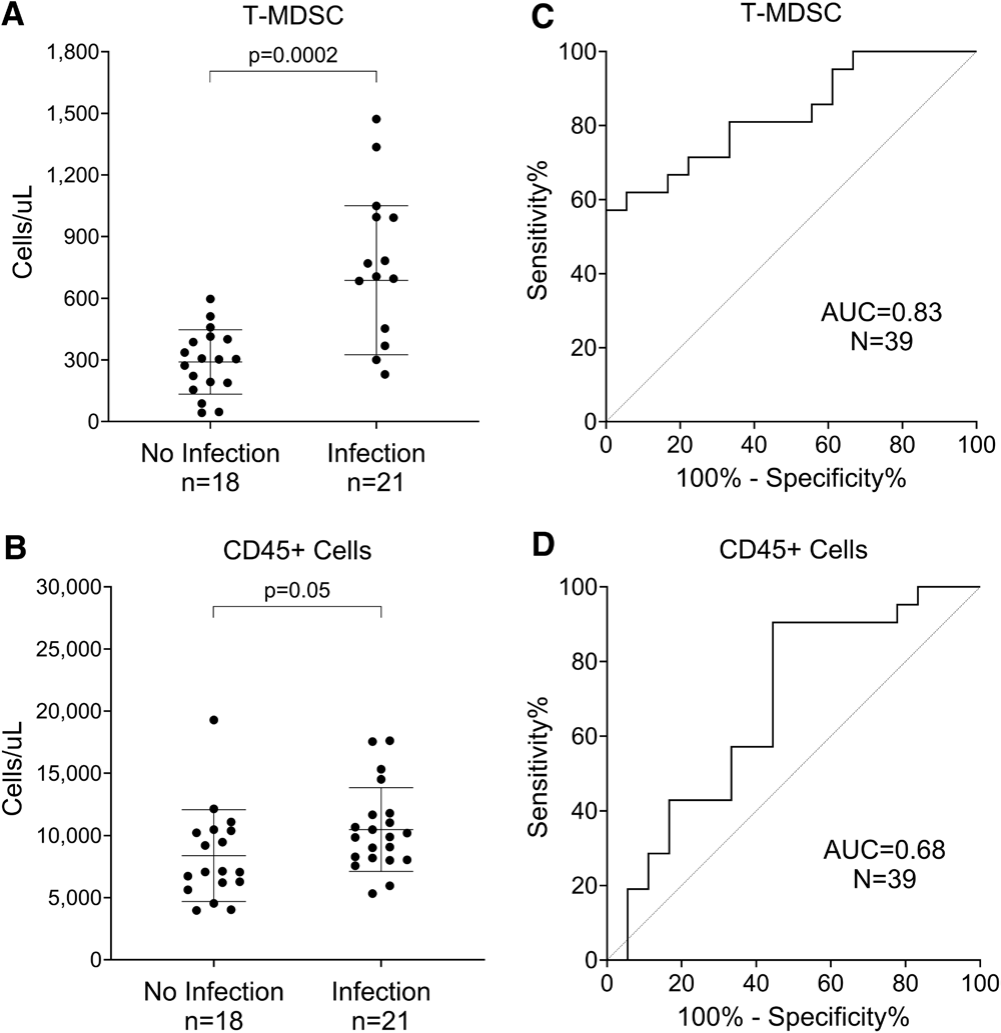
Comparison of total MDSC (T-MDSC) counts and CD45+ cell counts between subjects with and without infection. T-MDSC (**A**), and CD45+ leukocyte (**B**) counts (*y*-axis) from individual patients were measured using whole blood flow cytometry and are grouped by their infection status (*x*-axis). For the infected group, results obtained closest to their day of infection are plotted. Twenty of 21 infected patients had results before or on their infection day; the one exception had data obtained 1 day after infection. For the no-infection group (*n* = 18), the result shown is the first result obtained after hospitalization. *Bars* indicate the median with 95% CI for each group. *p* values were obtained using Mann-Whitney *U* tests. Receiver operating characteristic (ROC) curves for T-MDSC (**C**), and CD45+ leukocytes (**D**) measurements, using the same data points as in A–C, were plotted for outcomes of infection vs. no-infection. The area under curve (AUC) and total number of patients included in the analyses are shown.

Flow cytometry measurements of other immune cells (CD45+, lymphocyte, and neutrophil) were obtained from the same samples as the MDSC counts and all were compared between the groups (**Fig. 1**; and **eFig. 4**, http://links.lww.com/CCX/B474). T-MDSC counts were higher in subjects with infection than in those without (*p* = 0.0002), as were both subpopulations (polymorphonuclear-MDSC [*p* < 0.0002] and T-MDSC [*p* = 0.01]). CD45+ cells were used to represent WBC counts so that all analyzed measurements were generated using the same technology; CD45+ and WBC counts were highly correlated (Pearson *r* = 0.92, *p* < 0.001; **eFig. 5*A***, http://links.lww.com/CCX/B474). Although CD45+ cell counts were higher in patients with infection compared with no infection (*p* = 0.05), the values overlapped substantially **eFig. 5*B***, http://links.lww.com/CCX/B474). Among 21 infected patients, 12 had T-MDSC levels greater than 600 cells/µL at their timepoint closest to the day of infection. Of the nine infected patients with T-MDSC data below 600 cells/µL, four had data obtained 3–5 days before and one 2 days before the infection day.

We examined the performance of the T-MDSC, polymorphonuclear-MDSC, and CD45+ cell counts for differentiating those with infection from those without infection using ROC curves. The area under the ROC curve for T-MDSCs was 0.83 (95% CI, 0.70–0.96), whereas the CD45+ count had an area under curve of 0.68 (95% CI, 0.51–0.86) (Fig. 1, ***C*** and ***D***). The area under the ROC curve for polymorphonuclear-MDSCs was 0.84 (95% CI, 0.71–0.96) (data not shown).

## DISCUSSION

Severely injured trauma patients are an important subgroup of critically ill patients in whom the diagnosis of infection and sepsis may be particularly difficult, at least in part due to their co-existent response to injury. In this pilot study, we found that elevated T-MDSC and polymorphonuclear-MDSC levels were able to distinguish subjects with infection from those without, and in most cases, 1–2 days before the day infection was diagnosed; standard immune cell measurements such as CD45+ leukocyte or neutrophil counts did not. Previous studies have demonstrated associations between MDSC elevations and infection or sepsis but in most studies, MDSC levels were measured after the time of sepsis diagnosis (10, 14, 15). Our results indicate that MDSC measurements could be used to expedite microbiological testing for infection and additional decision-making.

Other biomarkers have been tested as tools for sepsis detection. Procalcitonin, for example, while useful for prognosis, is not sufficiently sensitive nor specific to guide diagnostic evaluation or initiation of treatment (16, 17). Monocyte distribution width (MDW) is another parameter that has been evaluated as a promising tool for early detection of sepsis (18). However, many clinical laboratories including ours do not report the MDW and we cannot compare our observations directly with this method. Novel biomarkers including gene expression panels such as the SepsisMetaScore (area under the ROC curve = 0.92) and SeptiCyte (Immunexpress, Seattle, WA) Laboratory (area under the ROC curve = 0.85) appear to accurately detect sepsis in critically ill patients (19, 20). These have not been widely adopted. Another method, the IntelliSep Index (ISI) is a proprietary test that quantifies physical changes in leukocytes and translates these into a sepsis risk index. The reported area under the ROC curve for the ISI is 0.83–0.91 (21, 22).

Our study has important limitations. First, this pilot study included 39 subjects from whom samples were obtained when laboratory staff and residual blood samples were available. This led to variability in the timepoints when blood samples were obtained after admission. The second potential limitation is that MDSC counts from subjects without infection were obtained earlier post-admission than in subjects with infection (median of 4 d vs. 6 d). This could lead to confounding if MDSC counts increase over time regardless of the presence or absence of infection. Studies of other biomarkers are also at risk of this potential confounding effect (17, 19, 23). Lastly, we did not compare our MDSC assay directly with other novel methods for infection or sepsis detection mentioned earlier. Although these limitations are important, they do not diminish the potential for using MDSC counts to guide infection management. Several steps remain, however, before recommending MDSC counts as a clinical aid (24, 25). For our part, we are performing a second study using the same enrollment criteria, but with more frequent blood sampling to help address the above-mentioned limitations.

## CONCLUSIONS

Quantitative assessment of whole blood T-MDSCs and polymorphonuclear-MDSCs using flow cytometry may aid in the earlier detection of infection in critically ill trauma patients.

## ACKNOWLEDGMENTS

We thank Scott Brakenridge, MD, for critical review of the data and article and Jin Wang, PhD, for statistical review and revision of the article.

## Supplementary Material
