# Supplementary material for: A Pilot Study Assessing the Utility of Quantitative Myeloid-Derived Suppressor Cell Measurements in Detecting Posttraumatic Infection
Source: Crit Care Explor. 2025 Mar 17;7(3):e1228. doi: 10.1097/CCE.0000000000001228 (PMC11918587; doi:10.1097/CCE.0000000000001228)
Supplement: Supplementary file 1 [file cc9-7-e1228-s001.pdf]

A Pilot Study Assessing the Utility of Quantitative Myeloid-Derived Suppressor Cell Measurements in Detecting Post-Traumatic Infection: Supplemental Digital Content

Table of Contents

	**STROBE Statement**	**Page 2 – 4**
**eMethods**	**Additional methods**	**Page 5 - 6**
**eFigure 1**	**Study participant inclusion flow diagram**	**Page 7**
**eTable1**	**Demographics and outcomes by infection category**	**Page 8**
**eFigure 2**	**Gating of CD45+ leukocytes, lymphocytes, neutrophils, and monocytes**	**Page 9**
**eFigure 3**	**Circulating cell counts from individual patients over time**	**Page 10 – 12**
**eFigure 4**	**Circulating MDSC levels vary significantly between infected and uninfected patients, while CD45+ leukocyte, lymphocyte and neutrophil counts do not**	**Page 13**
**eFigure 5**	**CD45+ cell counts obtained by flow cytometry and white blood cell (WBC) counts are highly correlated**	**Page 14**
**Citations**	**Additional citations**	**Page 15**

STROBE Statement—checklist of items that should be included in reports of observational studies

	Item No.	Recommendation	Page No.	Relevant text from manuscript
**Title and abstract**	1	(*a*) Indicate the study's design with a commonly used term in the title or the abstract	1 - title	
		(*b*) Provide in the abstract an informative and balanced summary of what was done and what was found	2	
		**Introduction**		
Background/rationale	2	Explain the scientific background and rationale for the investigation being reported	4	
Objectives	3	State specific objectives, including any prespecified hypotheses	4	We hypothesized that Total-MDSC counts (T-MDSCs) differ between subjects who develop infection and those who do not.
		**Methods**		
Study design	4	Present key elements of study design early in the paper	4	This single center, prospective observational cohort study….
Setting	5	Describe the setting, locations, and relevant dates, including periods of recruitment, exposure, follow-up, and data collection	4 and 5	
Participants	6	(*a*) *Cohort study*—Give the eligibility criteria, and the sources and methods of selection of participants. Describe methods of follow-up	4 – 5	
		(*b*) *Cohort study*—For matched studies, give matching criteria and number of exposed and unexposed	N/A	
Variables	7	Clearly define all outcomes, exposures, predictors, potential confounders, and effect modifiers. Give diagnostic criteria, if applicable	4 – 5	
Data sources/ measurement	8*	For each variable of interest, give sources of data and details of methods of assessment (measurement). Describe comparability of assessment methods if there is more than one group	4 – 5	
Bias	9	Describe any efforts to address potential sources of bias	7	
Study size	10	Explain how the study size was arrived at	5, 7	The sample size was not predetermined. We ended enrolment prior to linking clinical and outcome data with the flow cytometry data.

Continued on next page

	Item No.	Recommendation	Page No.	Relevant text from manuscript
Quantitative variables	11	Explain how quantitative variables were handled in the analyses. If applicable, describe which groupings were chosen and why	4 – 5	
Statistical methods	12	(*a*) Describe all statistical methods, including those used to control for confounding	5	
		(*b*) Describe any methods used to examine subgroups and interactions	N/A	
		(*c*) Explain how missing data were addressed	N/A	
		(*d*) *Cohort study*—If applicable, explain how loss to follow-up was addressed	N/A	
		(*e*) Describe any sensitivity analyses	N/A	
		**Results**		
Participants	13*	(a) Report numbers of individuals at each stage of study—eg numbers potentially eligible, examined for eligibility, confirmed eligible, included in the study, completing follow-up, and analysed	eFigure 1	
		(b) Give reasons for non-participation at each stage	eFigure 1	
		(c) Consider use of a flow diagram	Included – efigure 1	
Descriptive data	14*	(a) Give characteristics of study participants (eg demographic, clinical, social) and information on exposures and potential confounders	5, table 1	
		(b) Indicate number of participants with missing data for each variable of interest	N/A	
		(c) *Cohort study*—Summarise follow-up time (eg, average and total amount)	Table 1	
Outcome data	15*	*Cohort study*—Report numbers of outcome events or summary measures over time	Table 1	
Main results	16	(*a*) Give unadjusted estimates and, if applicable, confounder-adjusted estimates and their precision (eg, 95% confidence interval). Make clear which confounders were adjusted for and why they were included	5-6 Figure 1	
		(*b*) Report category boundaries when continuous variables were categorized	N/A	
		(*c*) If relevant, consider translating estimates of relative risk into absolute risk for a meaningful time period	N/A	

Continued on next page

	Item No.	Recommendation	Page No.	Relevant text from manuscript
Other analyses	17	Report other analyses done—eg analyses of subgroups and interactions, and sensitivity analyses		
		**Discussion**		
Key results	18	Summarise key results with reference to study objectives	6 – 7	
Limitations	19	Discuss limitations of the study, taking into account sources of potential bias or imprecision. Discuss both direction and magnitude of any potential bias	7	
Interpretation	20	Give a cautious overall interpretation of results considering objectives, limitations, multiplicity of analyses, results from similar studies, and other relevant evidence	7	
Generalisability	21	Discuss the generalisability (external validity) of the study results	7	
**Other information**				
Funding	22	Give the source of funding and the role of the funders for the present study and, if applicable, for the original study on which the present article is based	1	

*Give information separately for cases and controls in case-control studies and, if applicable, for exposed and unexposed groups in cohort and cross-sectional studies.

Note: An Explanation and Elaboration article discusses each checklist item and gives methodological background and published examples of transparent reporting. The STROBE checklist is best used in conjunction with this article (freely available on the Web sites of PLoS Medicine at http://www.plosmedicine.org/, Annals of Internal Medicine at http://www.annals.org/, and Epidemiology at http://www.epidem.com/). Information on the STROBE Initiative is available at www.strobe-statement.org.

eMethods

Additional information regarding the methods for subject identification and recruitment, infection assignment and details of the flow cytometry methods are included below.

Human subjects

This single center, prospective observational cohort study was approved by the University of Washington Institutional Review Board (STUDY00010131 [Immune Tests for Clinical Care; approval 5/27/2020] and STUDY00007918 [Protein-enhanced Enteral Nutrition and Metabolomics in Critically ill Trauma and Surgical Patients; approval 5/10/2021]). All study procedures were conducted in accordance with the ethical standards of the University of Washington Institutional Review Board and with the Helsinki Declaration of 1975. This study was classified as minimal risk by the IRB and study-specific informed consent was waived.

Subject identification and enrollment

Subjects were identified according to our previously described criteria. First, adult trauma patients receiving mechanical ventilation for ≥ 24 hours in the surgical ICU at Harborview Medical Center (Seattle, WA) were observed for possible enrollment. We excluded those who were not expected to survive due to the severity of their injuries, if they sustained a concomitant burn injury of ≥ 20% total body surface area, had chronic organ dysfunction, steroid or oncolytic use, were pregnant or lactating, or had HIV or COVID-19 infection. We then identified those who were intubated and receiving mechanical ventilation for at least 48 hours and were not expected to be extubated within the next 24 hours. Those who met this latter threshold were enrolled when laboratory staff were available to perform flow cytometry.

Infection and sepsis assignment

Infection and sepsis assignments, including the day of infection, were based upon review of medical records through 21 days after admission by two investigators (GO and NM) who were blinded to flow cytometry results. Sepsis assignments followed the Sepsis-3 guidelines.[1] For individuals categorized as infected (with or without sepsis), the likely source of infection was based upon review of the body fluid cultures and the clinical notes. Laboratory staff were blinded to clinical patient classification during testing. Six healthy subjects were enrolled as contemporaneous controls.

Flow cytometry

Remnant blood samples (i.e., blood that was obtained as part of clinical care and not specifically for this study) drawn in K_2EDTA vacutainer tubes (Becton Dickinson, Franklin Lakes, NJ) were analyzed within 4-12 hours of collection. We quantified circulating MDSC levels using a 9-color, 11-parameter flow cytometric assay.[2] Briefly, the following antibodies were applied to 100 uL of whole blood: CD45-V500-C (2D1), CD19-FITC (HIB19), CD20-FITC (L27), CD56-PECY7 (NCAM 16.2), HLA-DR-APC-H7 (L243), CD33-PE (WM53), CD11b-APC (ICRF44), and CD14-BV421 (MΦP9) (all, BD Biosciences); CD3-FITC(SK7), CD16-BV785 (3G8), and CD15-BV650 (W6D3) (all Biolegend). Total MDSC (T-MDSC) were defined as $CD45^+$ $CD3^-CD19^-CD20^-CD56^-CD16^-HLA\text{-}DR^-CD33^+$ $CD11b^+$ cells, while the monocytic (M-MDSC) and polymorphonuclear (PMN-MDSC) subsets were defined as $CD14^+$ and $CD15^+$, respectively. Absolute cell numbers were obtained using Trucount tubes (BD Biosciences). From the same sample, CD45+, neutrophil, and lymphocyte counts were obtained by flow cytometry; gating of these cell populations is shown in Supplemental eFigure 1. Data were acquired on a BD LSRFortessa cytometer and analyzed with FlowJo software v10.8 (Becton Dickinson, Ashland, OR).

eFigure 1. Study participant inclusion flow diagram.

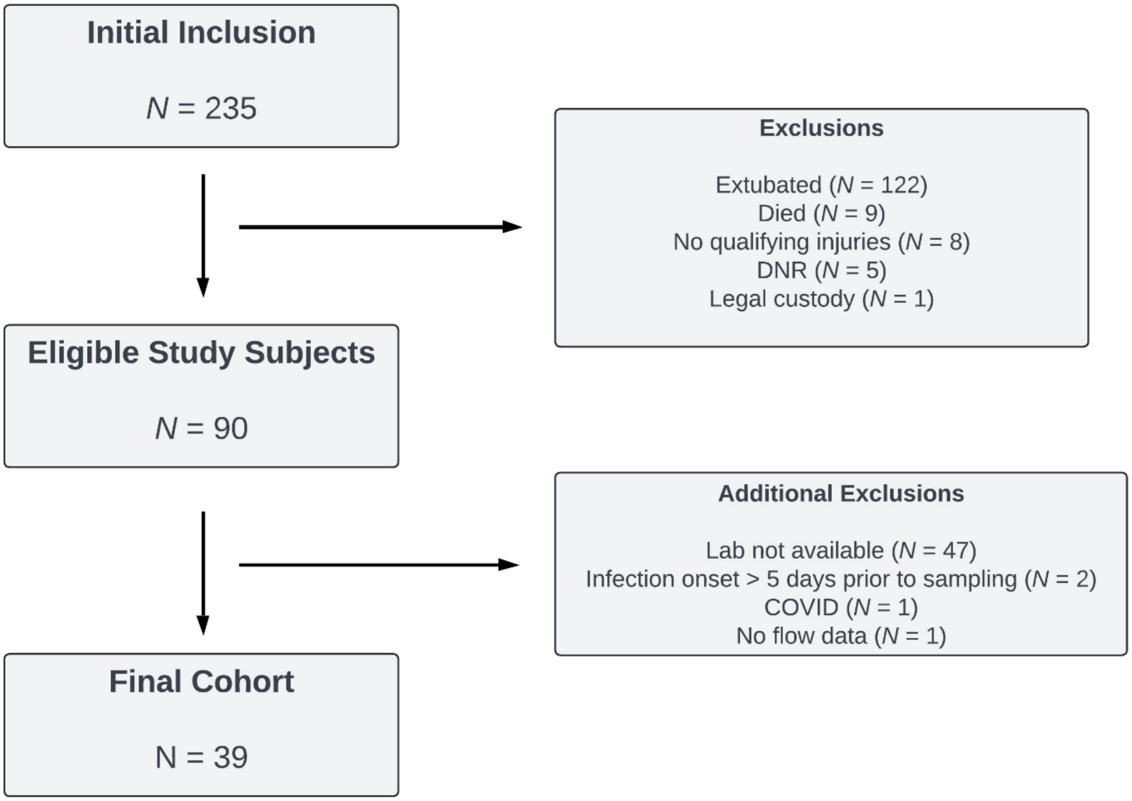

All potential subjects for study enrollment (Initial Inclusion) during the study period and reasons for exclusion are shown. Extubation was the primary exclusion criterion (n = 122). Of the eligible subjects, many were excluded due to unavailability of the laboratory staff (n = 47). The subject with no flow data had a sample collected, but technical problems with preparation rendered it unusable. One subject was diagnosis with SARS-2 COVID infection after enrollment and 2 subjects were diagnosed with infection > 5 days prior to any of their blood samples being obtained and these subjects were excluded from analysis.

eTable 1. Demographics and Outcomes by Infection Category

Characteristics	Infection (n = 21)	No Infection (n = 18)
Age, m (IQR, years)	46 (31, 66)	51 (35, 65)
Sex, n (%)		
Female	2 (10%)	6 (33%)
Male	19 (90%)	12 (67%)
Race, n (%)		
American Indian or Alaska	1 (5%)	2 (11%)
Asian	0 (0%)	2 (11%)
Black	3 (14%)	3 (17%)
White	15 (71%)	10 (56%)
Not documented	2 (10%)	1 (6%)
Mechanism of Injury, n (%)		
Blunt	18 (86%)	13 (72%)
Penetrating	3 (14%)	5 (28%)
Injury Severity Score, m (IQR)	33 (22, 41)	26 (20, 38)
Body Regions AIS≥3, n (%)[1]		
Head	10 (48%)	10 (56%)
Neck	4 (19%)	3 (17%)
Chest	15 (71%)	7 (39%)
Abdomen	6 (29%)	5 (28%)
Spine	10 (48%)	2 (11%)
Lower Extremity	7 (33%)	4 (22%)
Initial ED SBP <90mmHg, n (%)	4 (19%)	1 (6%)
RBC Transfusion First 24 hours, n (%)		
None	10 (48%)	11 (61%)
1-4 units	4 (19%)	7 (39%)
5-9 units	2 (10%)	0 (0%)
10+ units	5 (24%)	0 (0%)
Emergency Laparotomy, n (%)	6 (29%)	4 (22%)
Outcomes		
Hospital length of stay, m (IQR, days)	24 (19, 52)	18 (11, 41)
ICU length of stay, m (IQR, days)	17 (15, 22)	8 (5, 11)
Duration of Mechanical Ventilation, m (IQR, days)	18 (10, 19)	6 (5, 8)
Died (%)	6 (29%)	5 (28%)

Continuous data presented as median (IQR); discrete data as number (%).

[1]Body regions with frequency <10% are not shown which include face, upper extremity, and external injuries.

Abbreviations: Red blood cell (RBC), intensive care unit (ICU), length of stay (LOS).

eFigure 2

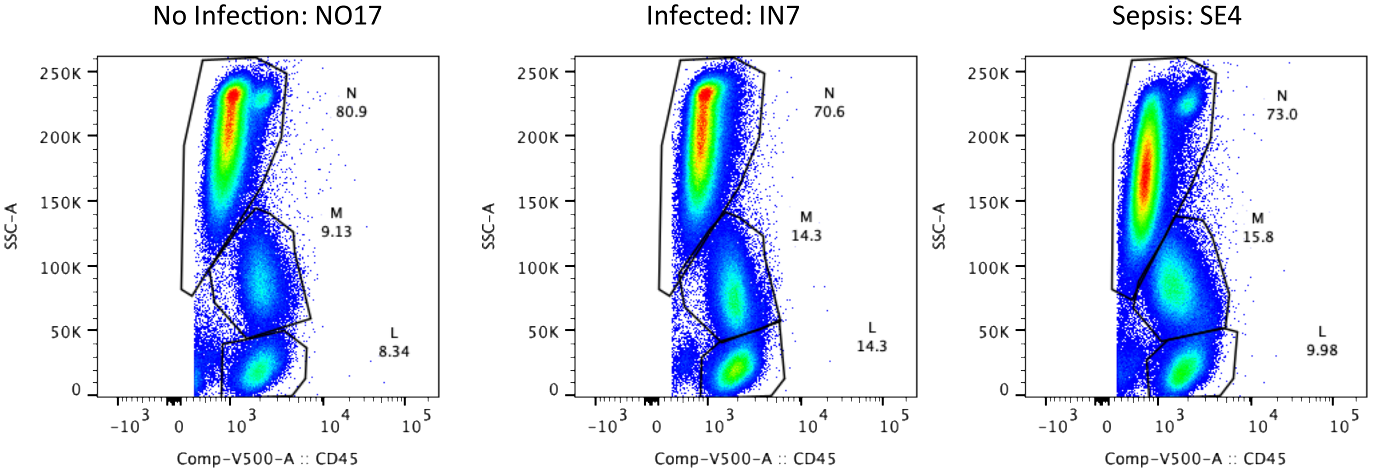

eFigure 2: Gating of CD45+ leukocytes, Lymphocytes, Neutrophils, and Monocytes.

Examples of cell populations comprising the CD45+ leukocyte population used in manuscript figures and tables. The CD45+ gate was defined after cell debris was removed using forward scatter area and side scatter area; Trucount beads were excluded using FITC and APC, and only single cells were selected using forward scatter height and forward scatter area. Neutrophil, monocyte and lymphocyte gates were defined by their expression of CD45, and side scatter area as shown above (N=neutrophils, M=monocytes, L=lymphocytes).

eFigure 3

A

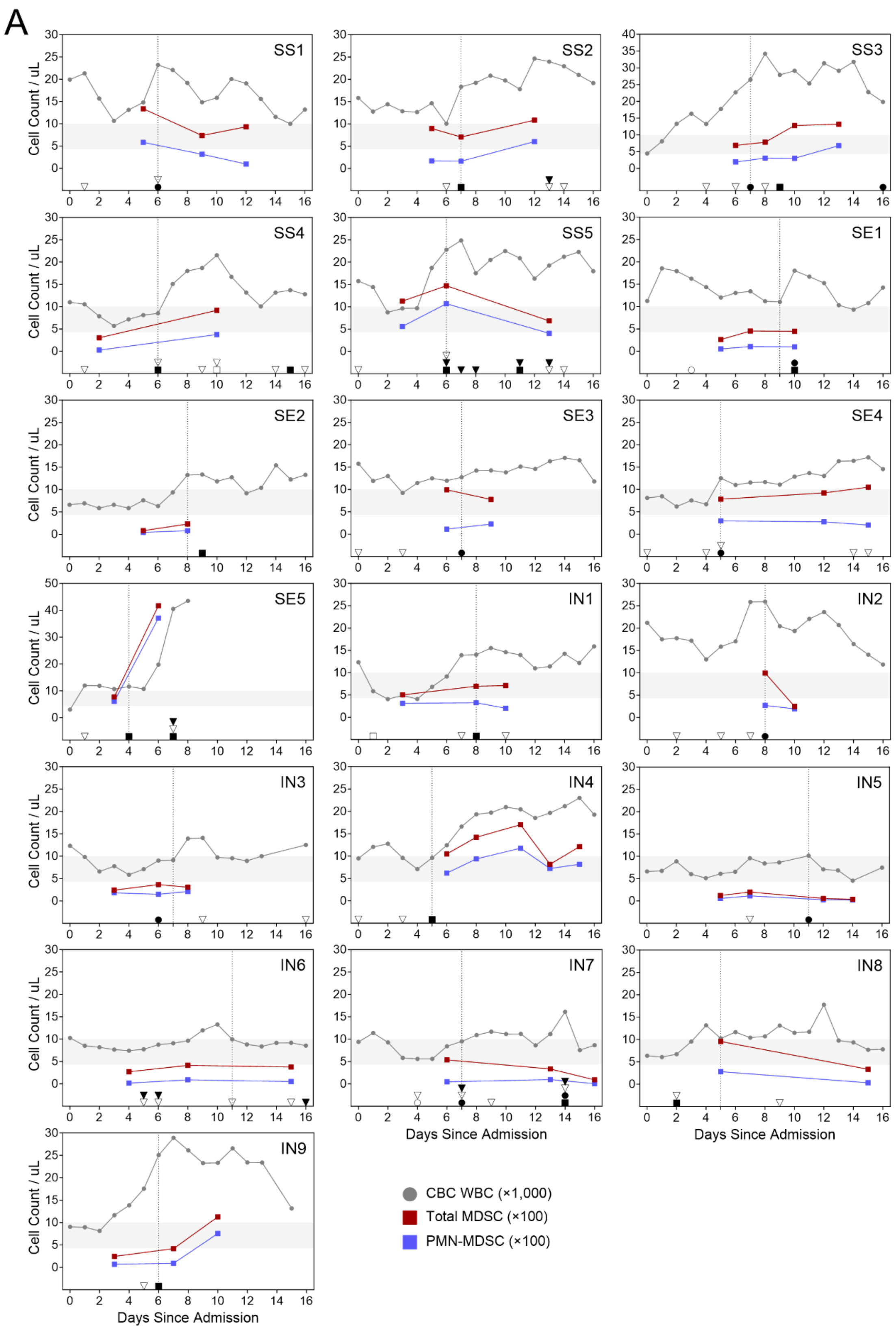

SS1
SS2
SS3
SS4
SS5
SE1
SE2
SE3
SE4
SE5
IN1
IN2
IN3
IN4
IN5
IN6
IN7
IN8
IN9
Cell Count / uL
Days Since Admission
CBC WBC (×1,000)
Total MDSC (×100)
PMN-MDSC (×100)

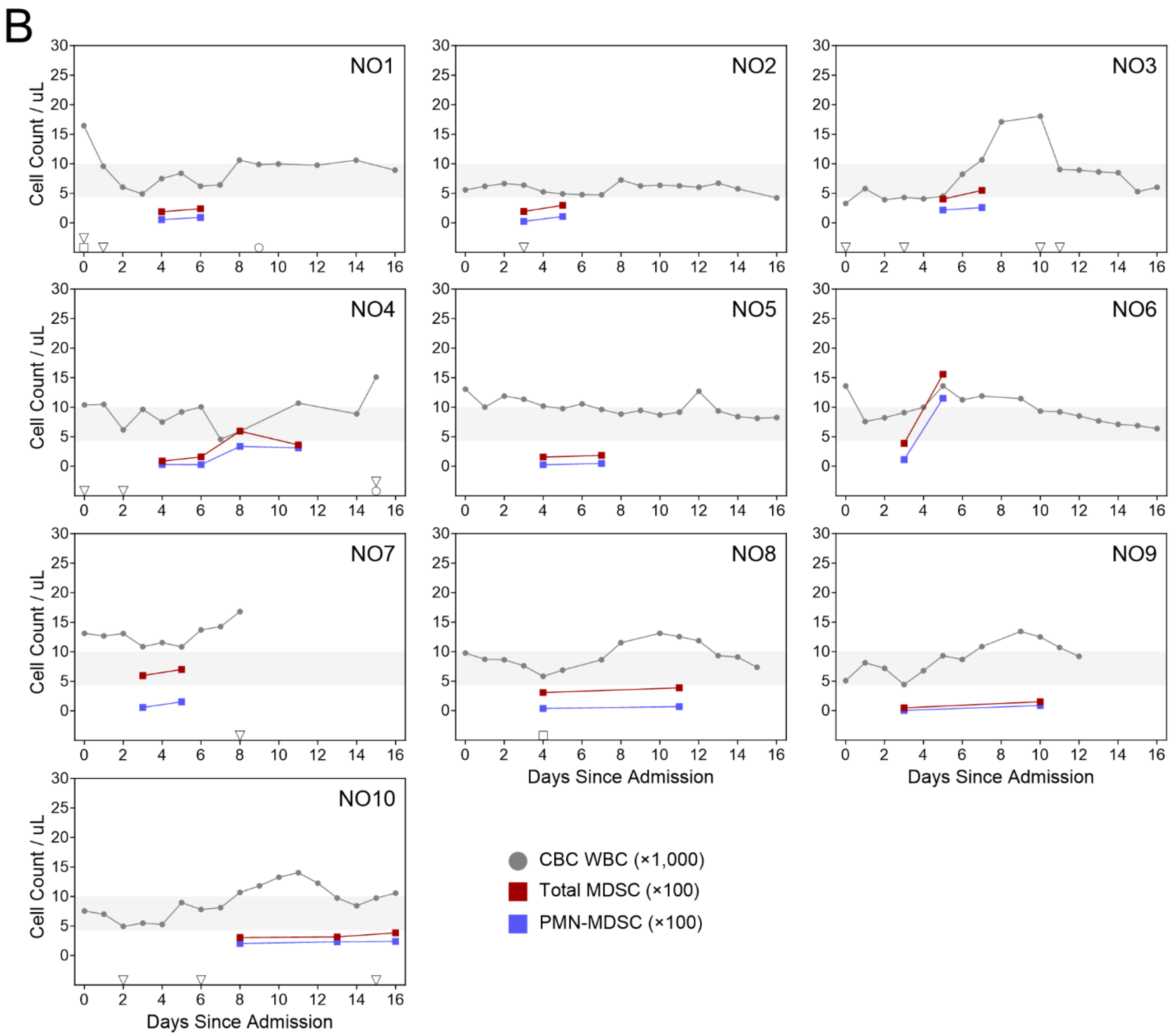

eFigure 3. Circulating cell counts from individual patients over time.

To understand the overall trends in MDSC counts after admission, we analyzed serially obtained blood samples when possible. A total of 29 of the 39 subjects had 2 or more samples tested, starting at day 2 or later after admission (median day 4). T-MDSC and PMN-MDSC counts over time were plotted and displayed in the context of clinical leukocyte measurements obtained daily (eFigure 2). The initial total MDSC counts for all 39 subjects (median 369 cells/uL) was higher than our previously established threshold for healthy volunteers of 110 cells/uL.

White blood cell (WBC) counts from Complete Blood Counts (CBC, gray), total MDSC counts (T-MDSC, red), and polymorphonuclear MDSC counts (PMN-MDSC, blue) are plotted against time (x-axis) for each individual patient. Patient IDs are indicated in the upper right of each panel, where prefixes indicate clinical categories: SS (septic shock), SE (sepsis), IN (infection) and NO (no-infection). The gray zone indicates the normal clinical reference interval for the WBC count, while the vertical dotted line indicates the infection day. Samples sent for investigation of possible infection are indicated by symbols at the bottom of each patient panel: bronchoalveolar lavage (BAL) culture (squares), endotracheal tube (ET) aspirate culture (circles), and other cultures (blood, urine, wound, etc.; triangles) according to the day of collection. The solid and open symbols indicate positive and negative results respectively. If multiple cultures were obtained on the same hospital day and all were negative, then only one symbol was used. If one of multiple cultures was positive, an additional solid symbol is shown to represent the positive culture. BAL and ET tube aspirates are all shown separately. Patients with only single timepoints tested are not shown in this figure (n = 9).

eFigure 4

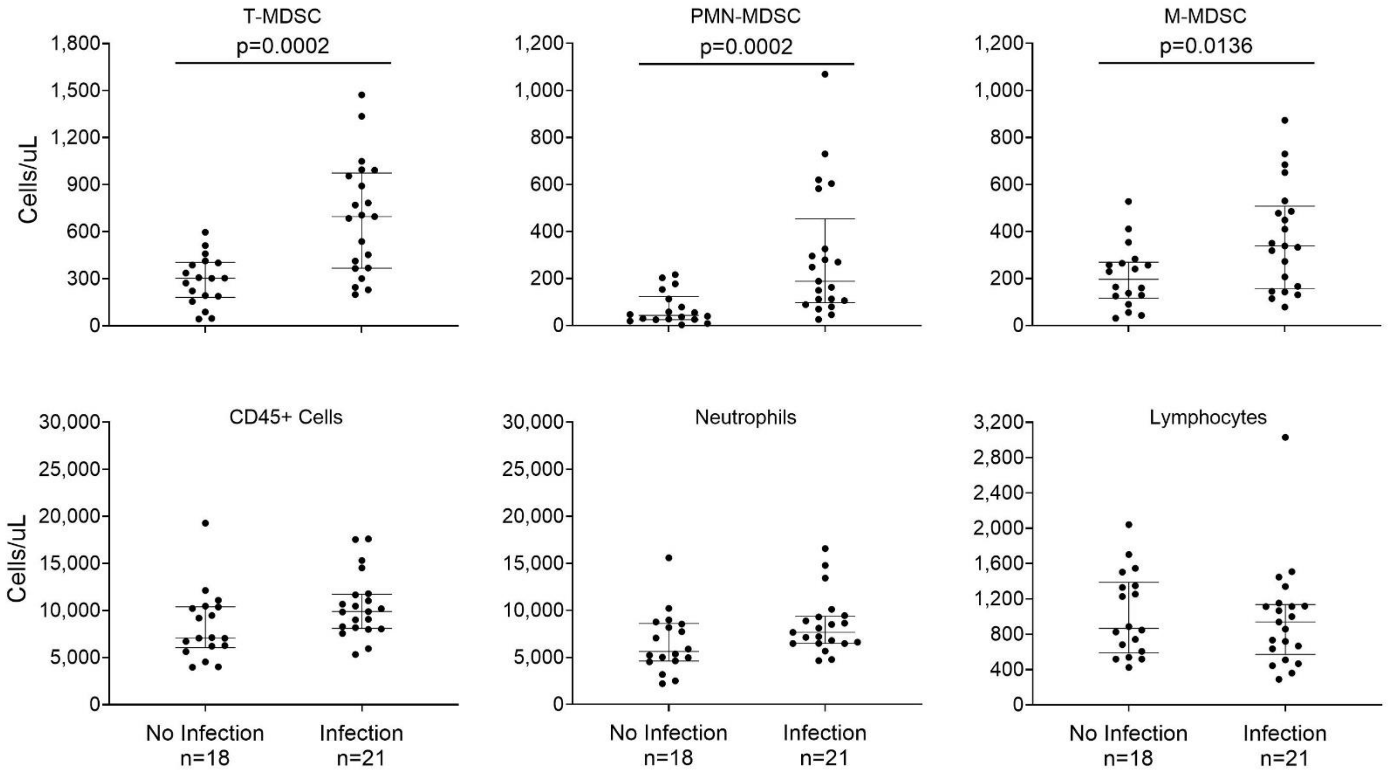

eFigure 4. Circulating MDSC levels vary significantly between infected and uninfected patients, while CD45+ leukocyte, lymphocyte and neutrophil counts do not.

Cell counts (y-axis) from individual subjects, grouped by their infection status (x-axis). For the infected group, results obtained closest to their date of infection are plotted. Twenty of 21 infected patients had results prior to or on their infection date; the one exception had data obtained 1 day after infection. For the no-infection group, the first result obtained during their ICU stay is shown. Bars indicate the median cell counts with the associated 25th – 75th percentiles. P values are from Mann-Whitney U tests. All data were obtained by flow cytometry as described in methods; CD45+ cells, neutrophils and lymphocytes were gated as shown in Supplemental Figure 1. MDSC were defined as $CD45^{+}$ $CD3^{-}CD19^{-}CD20^{-}CD56^{-}CD16^{-}$HLA-$DR^{-}CD33^{+}$ $CD11b^{+}$ cells, while the monocytic (M-MDSC) and polymorphonuclear (PMN-MDSC) subsets were defined as $CD14^{+}$ and $CD15^{+}$, respectively.

eFigure 5

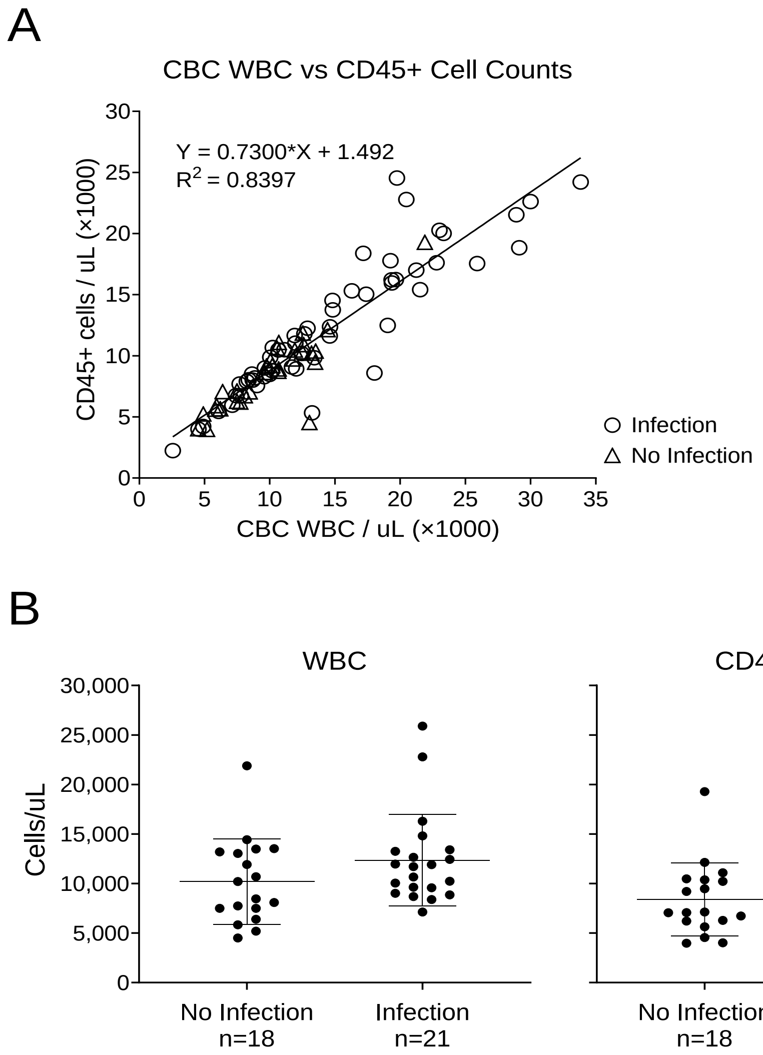

eFigure5. CD45+ cell counts obtained by flow cytometry and white blood cell (WBC) counts are highly correlated. CD45+ cells (gated as shown in Supplemental Figure 1, y-axis) are plotted against WBC counts (x-axis) obtained as part of a clinical complete blood count panel. Cell counts from all patients and from all timepoints when flow cytometry data were obtained are displayed (A). WBC or CD45+ cell counts, from the same timepoints used in Figure 3, are plotted according to their infection status (x-axis, B). Bars indicate the median cell counts with the associated 25th – 75th percentiles. Samples obtained closest to the date of infection were used for those diagnosed with infection, whereas the first samples obtained after hospitalization were used for those without an-infection.

Citations

1. Singer M, Deutschman CS, Seymour CW, et al. The Third International Consensus Definitions for Sepsis and Septic Shock (Sepsis-3). *JAMA*. Feb 2016;315(8):801-10. doi:10.1001/jama.2016.0287
2. Apodaca MC, Wright AE, Riggins AM, et al. Characterization of a whole blood assay for quantifying myeloid-derived suppressor cells. *J Immunother Cancer*. Aug 28 2019;7(1):230. doi:10.1186/s40425-019-0674-1
